# Editorial: Integration of NGS in clinical and public health microbiology workflows: applications, compliance, quality considerations

**DOI:** 10.3389/fpubh.2024.1357098

**Published:** 2024-01-23

**Authors:** Shangxin Yang, Varvara K. Kozyreva, Ruth E. Timme, Peera Hemarajata

**Affiliations:** ^1^Department of Pathology and Laboratory Medicine, David Geffen School of Medicine, University of California, Los Angeles, Los Angeles, CA, United States; ^2^Microbial Diseases Laboratory, California Department of Public Health, Richmond, CA, United States; ^3^Center for Food Safety and Applied Nutrition, United States Food and Drug Administration, College Park, MD, United States; ^4^Association of Public Health Laboratories, Silver Spring, MD, United States

**Keywords:** next-generation sequencing (NGS), clinical microbiology, public health microbiology, whole-genome sequencing (WGS), metagenomics, outbreak investigation, genomic surveillance, infectious diseases

Since its invention in the 90s, next-generation sequencing (NGS) has played an instrumental role in pushing our understanding of human microbiome and microbial genomics to a whole new level (1, 2). In the past decade, NGS has also been widely adopted in public health and food safety laboratories and became the primary method for microbial surveillance and outbreak investigation (3, 4). This trend extends to the clinical laboratories, where NGS has been a powerful tool for hospital outbreak investigation and institutional-level pathogen surveillance to aid infection prevention programs (5, 6). Soon after, the exploration of NGS's diagnostic utility for infectious diseases gained tremendous momentum, with both whole-genome sequencing (WGS) based and metagenomics (mNGS) based tests showing dramatic improvements in the detection and identification of pathogens that otherwise couldn't be detected or accurately identified, thus solving unmet clinical needs (7–9). Despite the great promise and indisputable values, integration of NGS is a challenging endeavor not only for each individual laboratory but also for our entire field. The technical complexity, the lack of guidelines and standards, and the extraordinary resources required are some of the most remarkable obstacles (10, 11). In this Research Topic, integration and utilization of NGS in clinical and public health microbiology was described in great detail, encompassing wet-lab techniques, bioinformatics, logistics, outbreak investigation, genomic surveillance, and patient diagnostics.

One highlight of this Research Topic is the use of fungal WGS for genomic surveillance, which historically had been less established compared to bacterial genomic surveillance. Using WGS, Michael et al. demonstrated that the infamous “Black Molds” epidemic during the delta wave of the COVID-19 pandemic in India was caused by multiple fungal species, predominantly *Rhizopus delemar*. Even among this species there was vast genetic diversity indicating no common sources nor a particular strain. Most COVID-19 patients who suffered mucormycosis had diabetes which is a known risk factor for both COVID-19 infection and invasive mucormycosis. These findings were significant as they demystified the role of Mucorales and its relationship with COVID-19. In another important study, Gorzalski et al. utilized 3 different bioinformatics tools to analyze the WGS data of over 200 *Candida auris* isolates from an ongoing large outbreak in Nevada and elucidated the inferred transmission networks based on shared SNP analysis. This study provided essential genomic epidemiological data to help understand the dynamics of this large outbreak which brought unprecedented challenges to the hospitals in the affected areas even to date. This study also highlighted the importance of implementing real-time genomic surveillance of *Candida auris* to help slow down its transmission.

The bioinformatics workflow TheiaEuk described in the manuscript by Ambrosio et al. was designed to fully utilize the benefit of a cloud computing platform. Their work on *C. auris* genomic epidemiological analysis involved a large dataset, which demonstrated that cloud computing is perhaps the only truly scalable and sustainable solution for bioinformatic analyses.

Analyzing fungal WGS data is challenging, yet the interpretation of fungal phylogenetic results can be equally hard, as demonstrated in another outbreak investigation by Fan et al.. In this study, *Cyberlindnera fabianii*, an unusual yeast was recovered from the urine culture of 3 patients from the same ward, prompting a suspicion for nosocomial infections. SNP analysis revealed that two of the *C. fabianii* isolates had 192 SNPs difference while the third was over 26,000 SNPs apart. The main conundrum was how to interpret this 192 SNPs distance; the authors did a literature review showing that the genetic difference of yeast isolates with epidemiological link could range widely from < 50 SNPs to >1000 SNPs, depending on the genome size of the species and length of the outbreaks. Given the similar size of *C. fabianii* compared to *C. auris*, 192 SNPs could still be interpreted as likely having a common source. Ultimately, the only way to solve this type of interpretation dilemma is to sequence many more fungal pathogens and pair it with extensive collection of epidemiological information on the potential transmission chains, which will expand the fungal genome database and our knowledge base of epidemiology of fungal nosocomial outbreaks and fungal evolution during infection and colonization.

The improvement in result turnaround time and increasing accessibility of sequencing technologies, even in limited-resources circumstances, allows researchers to find innovative ways to diagnose and improve the quality of care for high-consequence endemic diseases such as tuberculosis. In this Research Topic, we showcased seminal work by two different groups related to the use of targeted NGS (tNGS) for the diagnosis and prediction of antimicrobial resistance directly from primary specimens. tNGS approach for TB resistance prediction is clearly favorable for clinical application due to its ability to generate actionable results with a rapid turnaround time, as opposed to whole-genome sequencing, which is a great surveillance tool, but requires pure culture, hence, leading to delays in obtaining the results. The work by Murphy et al. described in detail a clinically validated, state-of-the-art approach to using tNGS coupled with real-time long read sequencing technology and customized bioinformatic pipeline to examine genes and mutations in *Mycobacterium tuberculosis* to predict resistance to antimicrobials, allowing clinicians to choose the most appropriate treatment for each patient weeks before WGS results were available. The work performed by de Araujo et al. further emphasized the potential feasibility of utilizing tNGS to enhance clinical and surveillance efforts to combat drug-resistant *M. tuberculosis* by outlining an innovative programmatic framework that incorporated *M. tuberculosis* tNGS in low-resource regions where NGS had not previously been available.

NGS has been widely used in surveillance of foodborne pathogens and healthcare associated pathogens, especially ones with resistant mechanisms of public health concern, like carbapenem-resistant organisms (CRO). Yet the utilization of genomic data for identification of outbreak sources and efficient communication of genomic results to the epidemiologists still could be improved to make genomic epidemiology truly actionable. Gali et al. highlights an application of automated, NGS cluster analysis tool at NCBI Pathogen Detection, which provides public health investigators current, pre-computed clustering data commonly used for the investigation of foodborne outbreaks. The Virginia Division of Consolidated Laboratory Services (DCLS) laboratory has extended this application to detect and identify the sources outbreaks of CRO, specifically involving *Acinetobacter baumannii, Enterobacter cloacae, Morganella morganii, Klebsiella pneumoniae, Escherichia coli*, and *Proteus mirabilis*.

The proper identification of pathogens is a cornerstone of successful surveillance as well as clinical diagnosis. However, even with such common pathogens as enteric bacteria, the scarcity of well-curated reference datasets impedes clinical validations of identification tools as well as the development of new bioinformatics solutions. The paper presented by Lindsey et al. described the development of Reference Genome Dataset for benchmarking of enteric genomic identification using Average Nucleotide Identity (ANI) algorithm. The manuscript also provides a nice example of clinical validation of a bioinformatics tool, including determination of genome coverage limits for successful ANI identification.

The mNGS technology has revolutionized pathogen detection. Historically, many fastidious or endemic pathogens have been under detected due to lack of effective diagnostic tools. As an agnostic “one-stop test,” mNGS is shown by Chang et al. to be particularly powerful in diagnosing a case of *Leishmania donovani* visceral leishmaniasis, a rare infectious disease unexpectedly found in an infant with acute lymphoblastic leukemia. The timely diagnosis led to successful treatment, demonstrating the value of mNGS. Another difficult-to-detect microorganism, *Legionella pneumonia*, was successfully identified with mNGS in a clinical case presented by Du et al. in which Legionnaires' disease coincided with rhabdomyolysis and acute kidney injury, a.k.a. Legionella Triad, a rare and deadly syndrome requiring timely diagnosis and treatment. The patient finally improved, and the authors advocate for the implementation of mNGS for the early diagnosis of severe cases of Legionnaires' disease in resource limited areas.

Question of clinical relevance of mNGS findings remains topical and matter of relative and absolute abundance of detected species and its importance in making clinical interpretations regarding the role of the detected pathogens as causative agents of the disease are widely discussed. Unlike aforementioned studies describing implementation of hypothesis-free mNGS approach, Almas et al. showcase the use of a hybridization capture-based sequencing method for the diagnosis of urinary tract infections (UTIs) by combining broad detection range benefits of NGS technology and precision of targeted approach for focusing data generation on clinically relevant information. The ability of the bioinformatic platform presented by the authors to provide quantitative results is particularly attractive for clinical microbiology applications.

Implementation of NGS is not without a set of unique challenges, which include but are not limited to requirement of expensive equipment and reagent, availability of skilled scientists to perform the wet lab part, access to high-performance computing platforms and well-trained bioinformaticians, and an effective way to validate and communicate results to clinicians and epidemiologists. Tartanian et al. astutely described not only their trials and tribulations in implementing SARS-CoV-2 sequencing within their health system, but also many aspects of their efforts that were incredibly successful. With the sheer volume of COVID-19 samples at the height of the pandemic, most, if not all entities performing SARS-CoV-2 sequencing had to find creative ways to increase the throughput of their sequencing efforts. One of the approaches taken by institutions with financial support was to implement automation throughout the NGS workflow in efforts to increase the throughput. Socea et al. described their success story, albeit not without some challenges, in implementing automated library preparation to eliminate one of the potential major bottlenecks in the NGS process. These stories of overcoming challenges to establish next-generation sequencing (NGS) capabilities provide valuable practical insights for those facing similar odds.

NGS, as a tool for pathogen genomic surveillance, requires tight coordination at multiple levels to ensure the NGS data are of sufficient quality and associated contextual data meet the requirements for public health action, both globally and locally. These coordinating efforts often include both public and private databases, increasing the complexity of data management in submitting and extracting data for public health action. Wadford et al. and the State of California established the California SARS-CoV-2 Whole Genome Sequencing (WGS) Initiative, or “California COVIDNet.” This cross-sector collaboration implemented large-scale genomic surveillance of SARS-CoV-2 across California to monitor the spread of variants within the state, to detect new and emerging variants, and to characterize outbreaks in various settings, including congregate facilities and workplaces. The framework and computational infrastructure developed for COVID-19 can be extended now for pathogen genomic surveillance of other infectious diseases.

Four decades ago, polymerase chain reaction (PCR) was invented and now it has become a primary diagnostic and screening tool for infectious diseases; NGS undoubtedly is following the same trajectory. Many efforts and innovations are still required to lower the cost and hurdles for the integration of NGS in clinical and public health microbiology, yet a bright future lays ahead. The advancements in automation, bioinformatics and database curation, and better consensus and guidelines for implementation of NGS assays in regulated environments for clinical testing will accelerate the widespread adoption of NGS and strengthen our capabilities for fighting the infectious diseases with ever-changing landscape.

## Author contributions

SY: Conceptualization, Writing—original draft, Writing—review & editing. VK: Conceptualization, Writing—original draft, Writing—review & editing. RT: Conceptualization, Writing—original draft, Writing—review & editing. PH: Conceptualization, Writing—original draft, Writing—review & editing.
